# Clinical significance of superior articular process as a reference point for free-hand pedicle screw insertion in thoracic spine

**DOI:** 10.1097/MD.0000000000009907

**Published:** 2018-02-16

**Authors:** Tae Hoon Kim, Suk Ha Lee, Jae Hyuk Yang, Jae Young Hong, Seung Woo Suh

**Affiliations:** a Department of Orthopedic Surgery, Konkuk University Medical Center, Konkuk University School of Medicine; b Scoliosis Research Institute, Department of Orthopedics, Korea University, Guro Hospital; c Department of Orthopedics, Korea University, Ansan Hospital, Korea.

**Keywords:** pedicle, perpendicular, superior articular process, thoracic spine, trajectory

## Abstract

The trajectory of the pedicle screw perpendicular to the SAP (superior articular process) is consistent with the universal trajectory presented in the previous study of the entry point using computed tomography. The ideal entry point and trajectory of pedicle screw insertion have been a matter of considerable debate. We attempted to find the relationship between SAP and entry point and trajectory of the pedicle screw.

Thoracic spine CT (computed tomography) scans of 9 volunteers were studied. A safe zone for the trajectory of the screw in the axial (Margin_Ax_) and sagittal (Margin_Sag_) was defined as the zone between lines perpendicular to the SAP along the medial and lateral cortex of the pedicle in the axial section, and the superior and inferior cortex in the sagittal section without violation of the pedicle walls. The midline of Margin_Ax_ and Margin_Sag_ was defined as the safe entry point of the trajectory in the axial and sagittal planes (Trajectory_Ax_ and Trajectory_Sag_), respectively.

Margin_Ax_ and Margin_Sag_ were 4.14 ± 0.99 and 9.03 ± 2.01 mm, respectively. There was a statistically significant difference in Trajectory_Ax_ between the upper and middle, and between the upper and lower (*P = *.0076 in both cases), but not between the middle and lower thoracic spine (*P = *.066). Trajectory_Sag_ was within 1 mm at the levels of T4, T8, T11 vertebrae and at 0 mm at the other levels. Thus, the midpoint of Margin_Sag_ was within 1 mm from the SAP base.

There was a constant angular relationship with the SAP and the pedicle axis; the line perpendicular to the SAP can act as a trajectory. Therefore, we suggest that the SAP might be the only accurate and safe reference for pedicle screw insertion in the thoracic spine perpendicular to the SAP using freehand technique.

## 1 Introduction

Pedicle screw fixation with the strong 3 column support has rapidly gained popularity as the posterior fixation of choice at the thoracic and lumbar spine in deformity correction and degenerative spine surgery.^[1]^ Unfortunately, mal-position of pedicle screw resulting in neurological and vascular complications is one of its most feared complications.^[2,3]^ To reduce the incidence of these complications, several insertion techniques such as freehand, fluoroscopically assisted, computer-aided, and open laminar K-wire assisted have been developed and the safety of these techniques have been evaluated with postoperative radiography and computed tomography (CT).^[2,4–10]^ Each of these techniques has its proponents and its own merits and demerits such as long learning curve in freehand technique, complexity in the computer-aided technique, radiation exposure in fluoroscopically assisted technique and additional bleeding, and a time-lag of open laminar K-wire assisted technique.^[8–10]^ Among these, freehand technique has been adopted at our scoliosis institution for pedicle screw fixation of thoracic and lumbar spine because its safety has been proven by several published studies.^[6,7]^ Moreover, it simplifies the process of pedicle screw insertion, speeds up surgical procedure, and there is no added radiation exposure risk.^[5]^ However, in practice the technique of inserting the pedicle screw into 3-dimensional vertebra should be considered in terms of both the entry point and trajectory. To the authors’ knowledge, there have been no published studies on the trajectory of a pedicle screw.

The initial idea of inserting the screw at the right angle into the superior articular process (SAP) came from reviewing postoperative CT scans of patients who underwent pedicle screw fixation using freehand technique through an ideal pedicle entry point.^[4]^ On reviewing previous cases, we found that pedicle screws inserted in the thoracic spine were nearly perpendicular to the SAP. This finding was similar to that by Roy-Camille and co-workers^[11]^ in 1986, who explained that the trajectory of the pedicle screw is essentially perpendicular to the posterior facet and parallel to the superior end plate in the sagittal plane, and to the midsagittal plate in the axial plane. Therefore, the SAP was chosen as the only landmark, and constant angle to the SAP (90°) was chosen as the angulation of the trajectory. However, the practical applications and the evidence for the SAP as the entry point have not been reported.

In this study, we investigated the relationship between the SAP and the trajectory of the pedicle in the sagittal and axial planes. Providing proof of a constant angular relationship between the pedicle and a specific anatomical landmark that does not change with thoracic levels should simplify pedicle screw insertion. If a constant anatomical relationship that allows safe pedicle screw insertion could be proven, this would potentially simplify the insertion techniques. A few previous studies have pointed to this fact.^[5,11,12]^

Theoretically, there will be a universally presenting trajectory when a pedicle screw is inserted in the thoracic spine using the freehand technique. We suggest that the trajectory of the pedicle screw perpendicular to the SAP might be consistent with the universal trajectory. We hypothesized that there is a constant angular relationship between the SAP and the axis of the pedicle, and that the line perpendicular to the SAP may serve as a trajectory. In addition, through CT evaluation, we verified that the trajectory of the pedicle screw perpendicular to the SAP is consistent with the universal trajectory presented in a previously reported study on the entry point and thoracic spine morphology.

## 2 Materials and methods

### 2.1 Study subjects

The inclusion criteria were defined so that patients with degenerative conditions were excluded from the study. After the approval by the Institutional Review Board (AFMC-10-IRB-012), 13 volunteers (five women and 8 men; 20–60 years old) with no history of trauma, fractures or metabolic bone diseases of the spine were included in the study. Anterior–posterior and lateral radiographs of the whole spine were taken to evaluate spinal deformity, fractures, and diseases such as infection, tumors, and metabolic disease. As a result, 4 patients were excluded, 1 because of scoliosis and 3 because of degenerative changes in the facet joint. The mean age of the remaining 9 volunteers was 30.6 years (range, 20–57 years) with an average height and weight of 173.9 cm (165–182 cm) and 70.8 kg (60–85 kg).

### 2.2 Radiological parameters for evaluation

The spatial relationship between the SAP and the pedicle was studied in 9 healthy volunteers by fine-slice CT to test the feasibility of safer free-hand pedicle screw insertion using the SAP as the only landmark. Computed tomography of the thoracic spine with a slice thickness of 1 mm was performed. All CT images and radiographs were analyzed by the Picture Archiving Communication System (LG Infinity, Seoul, Korea). On CT, several parameters were measured at each thoracic spine level, excluding the 12th thoracic vertebra (T12) level, as detailed below. The T12 was excluded because its SAP had a different shape than other thoracic vertebrae.

Radiological parameters were subdivided into 4 major groups.

**Figure 1:**
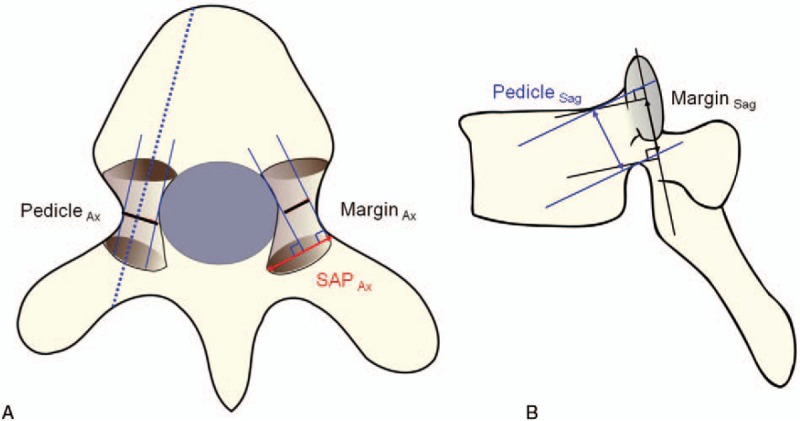
(A) Margin_Ax_ is the axial width of the safe border of the pedicle in the superior articular process when the screw is inserted at the right angle to the superior articular process (SAP), and is the shortest distance between perpendicular lines on the surface of the superior articular process in the axial plane that does not violate the medial and lateral pedicle walls. Pedicle_Ax_ is the axial diameter of the pedicle. Red arrowed line represents SAP_Ax_, the width of the SAP in the axial plane. (B) Black arrowed line represents Margin_Sag_, the sagittal width of the safe border of the pedicle and the shortest distance between perpendicular lines on the SAP surface in the sagittal plane that does not violate the superior and inferior pedicle walls. Blue arrowed line represents Pedicle_Sag_, the sagittal length of the pedicle. SAP = superior articular process.

**Figure 2:**
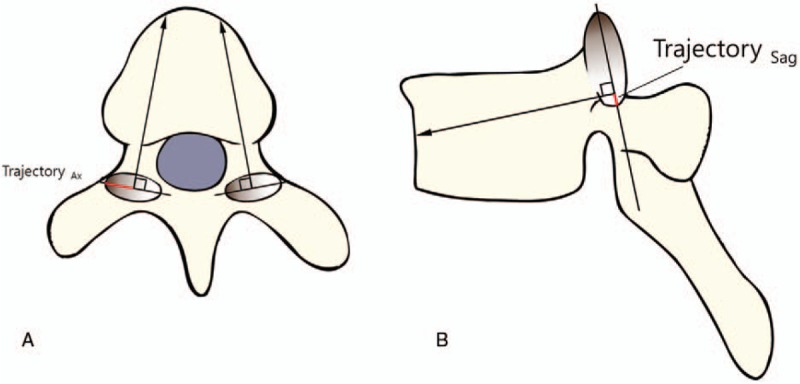
(A) The chord length in the axial plane (CL_Ax_) was measured as the distance from the superior articular process to the anterior wall of the vertebral body (black arrow); the red line is Trajectory_Ax_, which is the distance from the lateral margin of the superior articular process (gray ellipse) to the midpoint of Margin_Ax_. (B) The chord length in the sagittal plane (CL_Sag_) was measured as the distance from the superior articular process to the anterior wall of the vertebral body (black arrow). Trajectory_Sag_ was defined as the distance from the midpoint of Margin_Sag_ to the SAP base, and is represented as a red line. CL = chord length.

1. Morphometry of the SAP and pedicle size
  1. *Axial length of the SAP* was measured in millimeters (SAP_Ax_).
  2. *Pedicle size* was measured on the axial and sagittal slices of the isthmic portion (Pedicle_ax_ and Pedicle_Sag_).
2. *Safe margins (axial and sagittal)* for pedicle screw insertion: Axial and sagittal safe margins of the pedicle screw insertion in the SAP (Margin_Ax_ and Margin_Sag_) were measured as follows. On the axial slice, 2 lines were drawn perpendicular to the SAP surface along the medial and lateral borders of the pedicle so that the pedicle walls were not violated, and the shortest distance between them was measured in millimeters as Margin_Ax_. Similarly, on the sagittal slice, 2 lines were drawn perpendicular to the SAP along the superior and inferior borders of the pedicle so that the pedicle borders were not violated, and the shortest distance between them were measured in millimeters as Margin_Sag_ (Fig. 1A and B).
3. *Trajectory (axial and sagittal) for pedicle screw insertion:* The safe point of the trajectory (roughly interpreted as the ideal safe midpoint between the extremes of the margins) was identified on the axial and sagittal slices as Trajectory_ax_ and Trajectory_Sag_, respectively. Trajectory_ax_ was defined as the distance from the lateral SAP margin to the midpoint of Margin_Ax_. Trajectory_Sag_ was defined as the distance from the midpoint of Margin_Sag_ to the SAP base (Fig. 2).
4. *Chord length of the pedicle screw:* The chord length was measured on the axial and sagittal sections in millimeters (CL_ax_ and CL_Sag_): CL_ax_ is the length along the line perpendicular to the SAP, which joins the midpoint of Margin_Ax_ to the anterior cortex of the vertebral body in an axial cut (Fig. 2A). CL_Sag_ was the length along the line perpendicular to the SAP, which joins the midpoint of Margin_Sag_ to the anterior cortex of the vertebral body in a sagittal cut (Fig. 2B). Any violations of the pedicle wall by these lines were also noted.

**Figure 3:**
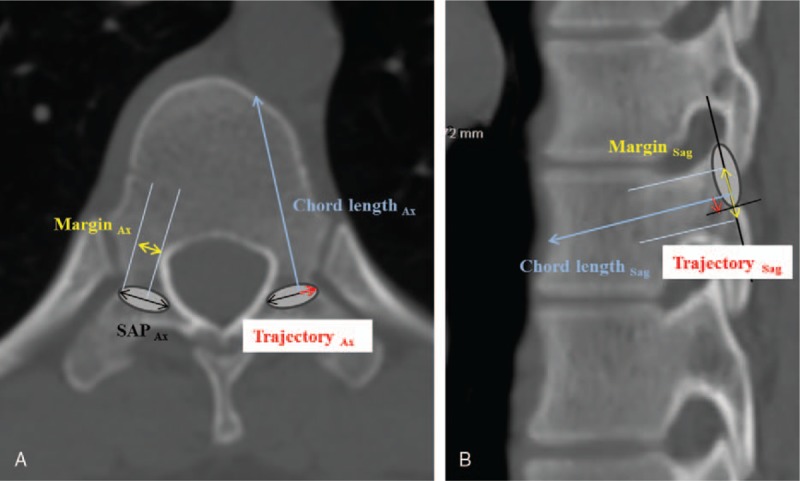
The axial (A) and sagittal (B) CT image showing the radiological parameters (yellow arrowed line: Margin_Ax_ and Margin_Sag_, blue arrowed line: CL_Ax_ and CL_Sag_, and red arrowed line: Trajectory_Ax_ and Trajectory_Sag_). CT = computed tomography.

The parameters were measured by 3 fellowship-trained orthopedic surgeons to test for interobserver reliability (Fig. 3A and B). Intraobserver reliability was measured by the analysis of the CT scans twice over a 3-week period with observer blinding.

### 2.3 Statistical analysis

The intra- and interobserver reliability was tested using the intraclass coefficient (ICC) test using the 2-way mixed and absolute agreement model. The ICC values were classified as follows: 0.8 to 1.00 as strongly reliable, 0.6 to 0.79 as reliable, and ≤ 0.59 as unreliable.^[13–15]^ The differences between the levels were analyzed by Wilcoxon signed-rank test. The entry point of the axial plane was analyzed as percentage of the axial width of the SAP; statistical significance of the differences was analyzed by Wilcoxon signed-rank test for the upper, middle and lower thoracic levels. The sagittal safety margin for the entry point related to the fixed-angled trajectory was analyzed by one-sample *t*-test. All statistical analyses were performed using SPSS Statistical Program, version 13.0 (SPSS, Inc, Chicago, IL). *P < *.05 was considered significant.

## 3 Results

We found excellent intra- and interobserver reliability for all radiological factors measured, as judged by the intraclass coefficient values of > 0.80 for all variables and by the mean values of 0.977 and 0.926 for intra- and interobserver reliability, respectively (Table 1).

**Table 1:**
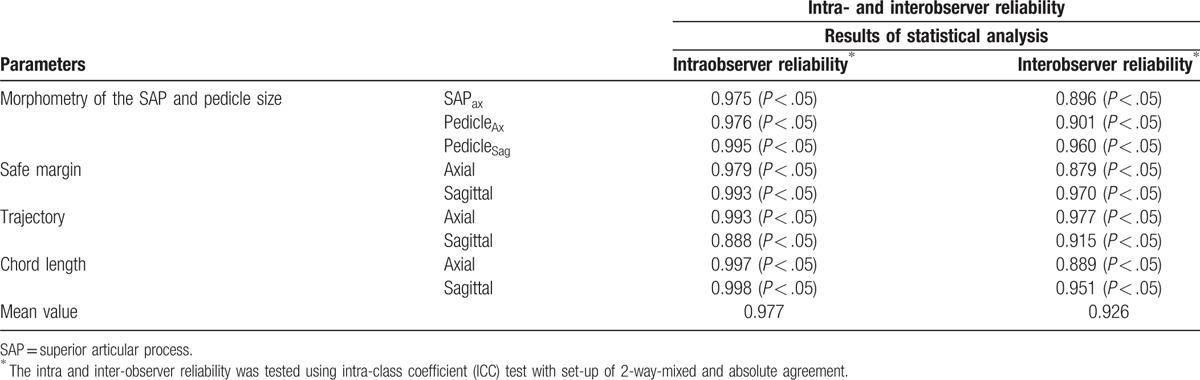
Intra- and interobserver reliability of radiological parameters.

The mean values of Margin_Ax_ and Margin_Sag_ were 4.14 ± 0.99 mm (range, 2.21–6.88 mm) and 9.04 ± 2.01 mm (5.01–15.65 mm), respectively. At the upper thoracic levels, the mean values of Margin_Ax_ and Margin_Sag_ were 4.86 ± 0.84 mm (range, 3.13–6.88 mm) and 7.44 ± 1.14 mm (5.37–9.8 mm), respectively. At the middle thoracic levels, the mean values of Margin_Ax_ and Margin_Sag_ were 3.52 ± 0.62 mm (range, 2.21–5.26 mm) and 9.06 ± 1.66 mm (5.01–12.84 mm), respectively. At the lower thoracic levels, the mean values of Margin_Ax_ and Margin_Sag_ were 4.94 ± 0.84 mm (range, 3.34–6.82 mm) and 11.34 ± 1.72 mm (8.21–15.65 mm). The mean values of Pedicle_Ax_ and Pedicle_Sag_ were 5.59 ± 1.09 mm (range, 2.95–9.78 mm) and 10.89 ± 1.84 mm (7.44–17.66 mm), respectively (Table 2). The mean value of Trajectory_Ax_ was 4.73 ± 2.05 mm (range, 0.91–9.81 mm) (Table 2).

**Table 2:**
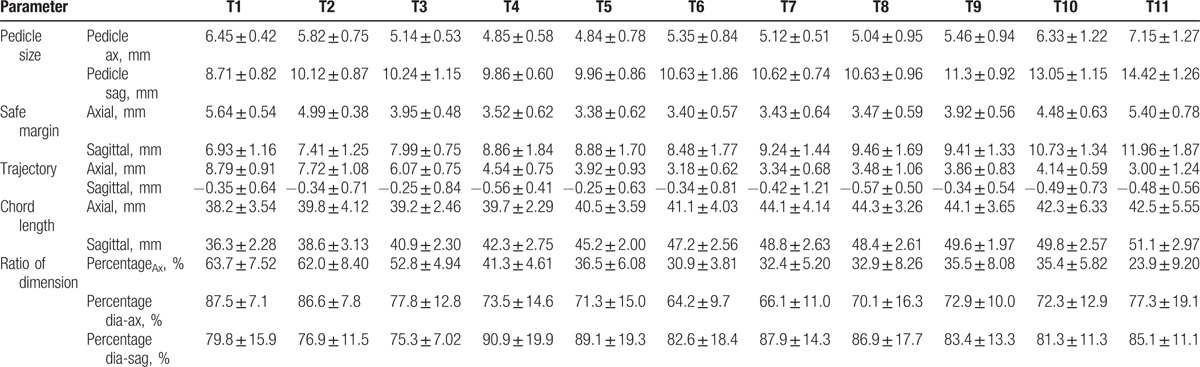
Measurement of radiological parameters in thoracic vertebra.

At the upper thoracic level, the mean value of Trajectory_Ax_ was 7.52 ± 1.45 mm (range, 4.86–9.81 mm). It was 3.72 ± 0.92 mm (1.57–5.92 mm) at the middle thoracic level, and 3.57 ± 1.12 mm (0.92 – 5.99 mm) at the lower thoracic level. Because the absolute values of Trajectory_Ax_ varied, the percentage of Trajectory_Ax_ relative to SAP_Ax_ was calculated (Percentage_Ax_). The mean value of Percentage_Ax_ was 40.7 ± 14.1% (range, 7.6–77.3%). At the upper thoracic levels, the mean value of Percentage_Ax_ was 59.5 ± 8.51% (43.3–77.3%). It was 34.9 ± 6.9% (16.8–50.6%) at the middle thoracic levels, and 29.7 ± 9.6% (7.6–49.1%) at the lower thoracic levels. The differences in Percentage_Ax_ between the upper, middle, and lower thorax were analyzed by Wilcoxon rank-signed test. Statistically significant differences were found between the upper and middle thorax, and between the upper and lower thorax (*P = *.0076 in both cases). There was no statistically significant difference between the middle and lower thorax (*P = *.66) (Table 3).

**Table 3:**
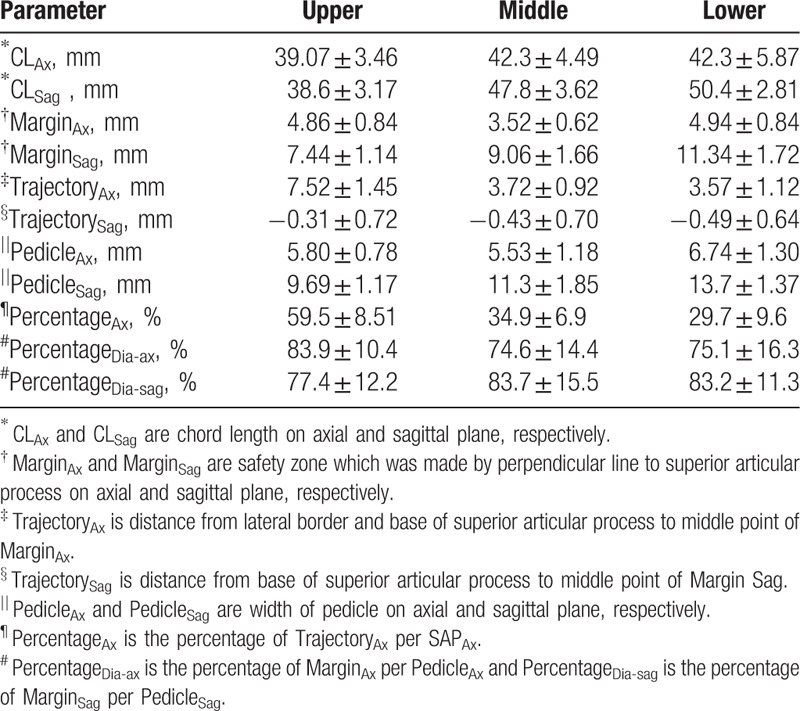
Parameters according to level of thoracic vertebrae.

The mean value of Trajectory_Sag_ was −0.4 ± 0.71 mm (range,−4.55–1.45 mm). The absolute value of this parameter was uniform and it was similar to the SAP base. Assuming that Trajectory_Sag_ was the same point of the SAP base, statistical analysis with one-sample *t*-test was performed. Statistical analysis showed that the mean value of Trajectory_Sag_ was 0 mm for T1, T2, T3, T5, T6, T7, T9, and T10 thoracic vertebrae (*P = *.12, .18, .39, .24, .19, .17, .07, and .07, respectively), and 1 mm for T4, T8, and T11 (*P = *.004, .006, and .001). CL_ax_ and CL_Sag_ were 41.4 ± 4.5 mm (range, 28.9–50.5 mm) and 45.3 ± 5.4 (30.3–55.4 mm), respectively, and none of the CL_ax_ and CL_Sag_ lines violated the pedicle walls.

## 4 Discussion

The SAP is a natural choice as an insertion point because of its anatomical continuity with the pedicle. We found SAP to be a constant anatomical landmark, which is present at each level and is useful for finding the proper entry point.^[4,6,7]^ Moreover, a proof of SAP reliability would eliminate the need in external references such as the relationship to the transverse process, and thus simplify the process and also set the stage for preparatory studies on cadavers and biomechanical aspects before its real-world adoption.

**Figure 4:**
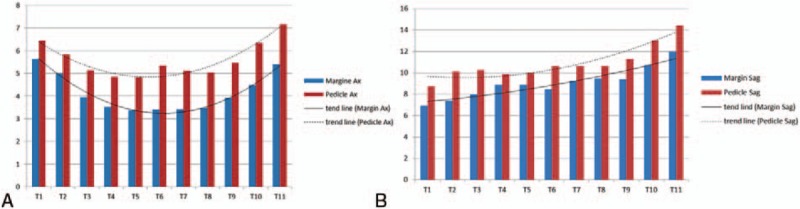
(A) The trend line of Margin_Ax_ showed same pattern as Pedicle_Ax_. This means that Margin_Ax_ properly reflects Pedicle_Ax_. (B) The trend line of Margin_Sag_ also showed the same pattern as Pedicle_Sag_.

Predictably, the value of the pedicle diameter also showed a similar tendency, as the axial diameter of T5 and the sagittal diameter of T1 were the smallest. This has also been observed in other studies into pedicle morphometry.^[16–19]^ This seems to indicate that Margin_Ax_ and Margin_Sag_ vary in proportion with the pedicle diameter (Fig. 4A and B). For additional confirmation, we evaluated the percentage ratio between Margin_Ax_ and the axial diameter of the pedicle (Percentage_Dia-ax_), and the percentage ratio between Margin_Sag_ and the sagittal diameter of the pedicle (Percentage_Dia-sag_). The values of Percentage_Dia-ax_ and Percentage_Dia-sag_ showed a linear pattern even though there is little difference between the upper and other thoracic levels due to the anatomical difference.^[20]^ The mean values of Percentage_Dia-ax_ and Percentage_Dia-sag_ from T1 to T3 were 83.9 ± 10.4% and 77.3 ± 11.9%, respectively, and were constant. The mean values of Percentage_Dia-ax_ and Percentage_Dia-sag_ were also constant from T4 to T11 and were evaluated as 71.1 ± 14.2% and 85.9 ± 15.9%, respectively. These results indicate that Margin_Ax_ and Margin_Sag_ also constantly reflect the diameter of pedicle in all thoracic vertebrae. Consequently, the Margin_Ax_ and Margin_Sag_ values are safe enough to rely on when inserting the awl for the entry point, curved gearshift for the second probing and pedicle screws.

If this method of screw insertion is to be adopted, it should ensure determination of the proper length of the screw to be introduced in order to purchase screws of adequate dimensions. The chord length is a key determinant in this respect. The mean chord length was 41.4 ± 4.5 mm in the axial plane (CL_ax_) and 45.3 ± 5.4 mm in the sagittal plane (CL_Sag_). These results are similar to 38.9 to 46.6 mm for T4 to T12 suggested by Vacarro et al^[16]^ and to 35.7 to 45.5 mm for T1 to T12 suggested by Zindrick et al^[21]^ as CL_Ax_. This means that the chord length estimated from the fixed-angle trajectory was comparable.

**Figure 5:**
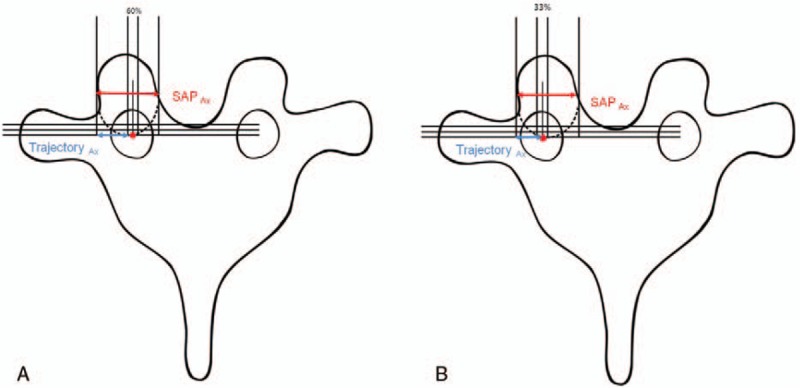
(A) At the upper thoracic level, an ideal entry point is located at 60% of the axial length of the superior articular process (SAP_Ax_, red arrowed line) in the axial plane and on the inferior border of the superior articular process in the sagittal plane. (B) At the middle and lower thoracic levels, ideal entry points are located at 33% of the axial length of the superior articular process (SAP_Ax_, red arrowed line) in the axial plane and at the inferior border of the superior articular process in the sagittal plane. SAP = superior articular process.

The entry point and trajectory described here are easy to adopt as they are fixed across various thoracic levels. Previous reports have described the introduction of pedicle screws using the relationships between the superior articular process and the transverse process, or between the inferior articular process and the transverse process.^[5,11,12,16]^ These procedures can be simplified using only SAP as anatomical landmark, and Trajectory_Ax_ and Trajectory_Sag_ were used as scales to localize the entry point on the axial and sagittal planes of the SAP.^[6,22]^ The mean value of Trajectory_Ax_ was 4.73 ± 2.05 mm, but the absolute value of Trajectory_Ax_ varied according to the SAP width at each thoracic vertebra. Therefore, the percentage ratio based on the transverse length of the SAP (Percentage_Ax_) was used to avoid using the absolute value, which could complicate the calculations. In the analyses of Percentage_Ax_, its values were consistent within 2 separate intervals, from T1 to T3 and from T4 to T10. The cause of this pattern has been suggested to be the anatomical difference in vertebral shape between the upper and other levels.^[20]^ This finding was also confirmed statistically (*P = *.0076, .0076, and .066). The mean value of Percentage_Ax_ for the upper thoracic vertebrae was 59.5 ± 8.51%, and the mean value of other thoracic vertebrae was 33.6 ± 8.00% (Fig. 5A and B).

The mean value of Trajectory_Sag_ was measured as −0.4 ± 0.71 mm, and was relatively constant regardless of the level of thoracic vertebrae. The value of Trajectory_Sag_ was within 0 mm for T1, 2, 3, 5, 6, 7, 9 and 10 (*P = *.12, .18, .39, .24, .19, .17, .07, and .07), and within 1 mm for T4, 8 and 11, and was statistically significant (*P = *.004, .006, and .001). This result is similar to the data of Roy-Cammille and co-workers,^[11]^ who described the pedicle entry point as 1 mm below the facet joint. Considering that the mean value of Margin_Sag_ was 9 mm, the difference of 1 mm is very small and may have little practical significance. Therefore, if the entry point is at the SAP base, the line made from this point at a 90 degree angle to the SAP will be mostly safe without violating the pedicle wall.

From these results, we conclude that if the 90° fixed-angle line is used as a trajectory, the entry point in the axial plane will be located approximately at 60% of the SAP width at T1–T3, and at 33% at T4–T11, and at the SAP base sagittally (Fig. 5A and B). The pedicle screw inserted at this point at the right angle should not violate the pedicle walls in both axial and sagittal planes.

This study has some limitations. First, the SAP is not always a constant anatomical landmark in all scoliosis patients. However, the SAP is a natural choice as an insertion point because of the anatomical continuity with the pedicle. Further investigations, including cadaveric studies, will be necessary for a more accurate evaluation of the entry point, trajectory and safe margin for free-hand pedicle screw insertion in the thoracic spine. Second, thoracic pedicle screws are indispensable for surgical treatments in such disease as scoliosis, ossification diseases, fracture, dumbbell shaped spinal cord tumor, and metastatic tumors. However, only normal populations were included in this study. Since this is the first study, we have naturally undertaken this on subjects with normal spine. It only stands to reason that the theory be tried out and proven safe in a group of subjects with normal anatomy before testing it on the scoliosis population with abnormal anatomy. In practice, we previously reported good accuracy and safety of pedicle screw placement in idiopathic and neuromuscular scoliosis performed by freehand technique using SAP entry point.^[6,7]^ SAP reference point using freehand technique might be applied carefully in a group of subjects with abnormal anatomy. To overcome these limitations, further research of thoracic pedicle screw placement in various diseases will be necessary.

## 5 Conclusion

We demonstrated a constant angular relationship between the SAP and the pedicle axis, and found that the line perpendicular to the SAP can act as a trajectory. Therefore, we suggest that the SAP may be the only accurate and safe reference for pedicle screw insertion in the thoracic spine perpendicular to the SAP using the freehand technique.
